# Reconstruction of the coracoacromial ligament during a modified Latarjet procedure: a case series

**DOI:** 10.1186/s12891-015-0698-8

**Published:** 2015-09-04

**Authors:** Matthias Aurich, Gunther O. Hofmann, Florian Gras

**Affiliations:** Department of Orthopaedics and Trauma Surgery, Sana Kliniken Leipziger Land, Sana Klinikum Borna, Rudolf-Virchow-Str. 2, 04552 Borna, Germany; Department of Trauma, Hand and Reconstructive Surgery, University Hospital Jena, Erlanger Allee 101, Jena, 07747 Germany

## Abstract

**Background:**

The coracoacromial ligament (CAL) is an important restraint to superior shoulder translation. CAL release with the Latarjet procedure leads to increased superior humeral translation. Therefore, a surgical technique was developed to reconstruct the CAL during a modified Latarjet procedure.

**Methods:**

Between May 2010 and July 2011, six patients (five were male, one was female; age 23–41 years) with chronic post-traumatic anterior shoulder instability were treated surgically with a modified congruent-arc Latarjet procedure (modLAT) with additional reconstruction of the CAL using a newly developed procedure, the pectoralis minor fascia flap (PMFF). Clinical follow-up was performed for up to 36 months, and patients were evaluated using a Rowe score.

**Results:**

All six patients experienced chronic, post-traumatic anterior shoulder instability and had experienced multiple re-dislocations after initial treatment. The preoperative assessment showed a defect of the anterior glenoid in three cases, and the mean Rowe score was 16.67 (5–25). Open modLAT with PMFF resulted in a stable shoulder function with no re-dislocations. The Rowe score increased from 77.5 (65–90) at 12 weeks to 95 (90–100) at 12 months and plateaued thereafter. Operative duration was 95 min (78–112 min), and there were no intra- or postoperative complications. All patients returned to their preoperative sports activity, three at the same level.

**Conclusion:**

The PMFF is a safe technique for reconstruction of the CAL during a modLAT procedure. Patients had improved shoulder function and no re-dislocations after the surgery.

## Background

The function of the coracoacromial arc—specifically, of the coracoacromial ligament (CAL)—in superior shoulder stability has been well established [1–5]. Along with the acromion, the inferior, concave surface of the CAL acts as a static restraint to superior translation of the humeral head. Conceptually, with CAL resection in the setting of a large rotator cuff tear or previous surgery, the humeral head may become prone to anterosuperior migration [6, 7]. In light of this, more attention has been focused on the CAL to define its role in glenohumeral joint stability [8].

*In vitro* biomechanical studies have been performed to illustrate the role of the CAL in superior stability. These studies have demonstrated increased superior translation with varying forces applied to the shoulder following CAL resection [1, 2, 4, 6, 7]. As a result, it was advocated to maintain the integrity of this structure whenever possible to avoid destabilizing the glenohumeral joint. There is also a relationship between CAL resection and anterior glenohumeral instability. An intact CAL is thought to interact with the coracohumeral ligament to provide restraint to anterior and inferior translation, as CAL resection has been shown to result in increased anteroinferior instability, indicating that its role in shoulder stability is larger than historically presumed [1, 2].

Post-traumatic anteroinferior glenohumeral instability is a common clinical entity. The Latarjet procedure (*Latarjet*), which involves a transfer of the coracoid process (CP) along with the conjoined tendon, is an attractive surgical option for the management of anterior shoulder instability in the setting of bony defects [9, 10]. In the treatment of substantial anterior glenoid bone loss, the CP restores the width of the glenoid to prevent further instability [10–14]. Additionally, the dynamic sling effect of the conjoined tendon is thought to enhance the stability provided by the bone block alone [10].

Classic Latarjet [9] involves transfer of the coracoid body with fixation of its inferior surface to the anterior glenoid vault. Recently, a congruent-arc modification of the Latarjet has been described, in which the coracoid bone block is rotated 90° so that its inferior surface is flush with the glenoid articular cavity [13]. The congruent-arc Latarjet has been reported to provide a better match of radius of curvature to the native glenoid [11], better normalization of glenohumeral contact pressures, [15] and the ability to reconstitute a larger glenoid bone defect than a CP oriented in the classic manner, thus theoretically improving anteroinferior stability [11, 15].

The Latarjet coracoid transfer has been reported to be largely successful, with re-dislocation rates as low as 4.9 % after 5 years and good to excellent functional outcomes [12, 14]. However, both the classic and the congruent-arc Latarjet transfers involve separation of the CAL. Therefore, we developed a novel surgical technique to reconstruct the CAL using a pectoralis minor fascia flap (PMFF) with a modification of the congruent-arc Latarjet (modLAT). This report describes the technique in detail and presents the results of a small case series.

## Methods

### Patients

The study was performed with the approval of the Ethics Committee of the University of Jena (No. 4176-08/14) and carried out in compliance with the Helsinki Declaration.

Six patients with chronic post-traumatic anterior shoulder instability and recurrent anteroinferior shoulder dislocations were surgically treated with a novel modification of the open Latarjet coracoid transfer (modLAT). As part of the procedure, the CAL was reconstructed with a fascia flap from the pectoralis minor muscle (*pectoralis minor fascia flap* [PMFF]).

Patients’ characteristics are summarized in Table 1. All patients had had multiple dislocations before the modLAT was performed. In addition, all patients had a history of a first traumatic shoulder dislocation and initial arthroscopic stabilization, patients 2–6 at outside hospitals. Patient 1 had his index dislocation at the age of 36 years, when he fell from a roof. After initial reduction, he was treated conservatively. Re-dislocation occurred following a fall from a ladder 3 years later. Instability developed, with re-dislocations several times per week with inappropriate movements of the arm. Patient 2 had his first dislocation after a cycling accident. Re-dislocation occurred when he jumped into a pool from a height of about 5 m. Patients 3, 4, 5, and 6 had the initial dislocation during their sports activities. Patients 3 and 6 had re-dislocations as a result of direct trauma (both while playing soccer). In patients 4 and 5, re-dislocations occurred during their sports activity, but without bodily contact. In patients 4 and 5, an open capsular plication procedure was performed, whereas patients 2, 3, and 6 underwent arthroscopic revision surgery. Despite these revision procedures, chronic instability with recurrent dislocations developed in all cases.

**Table 1:** Patient characteristics

Patient	1	2	3	4	5	6	Average
Gender	male	male	female	male	male	male	
Sports/Activity	soccer	cycling	canoeing	handball	swimming	soccer	
Shoulder-Dislocations	occasionally	occasionally	when canueing	daily	when swimming	occasionally	
Glenoid defect anterior	yes	no	no	yes	yes	no	
Age at Surgery [years]	41	23	25	23	24	28	27,33
Time of Surgery [min]	96	108	84	91	112	78	94,83
Time of Rehab [weeks]	6	6	6	6	6	6	
Return to Sports	yes	yes	yes	yes	yes	yes	
same level	x	x				x	
lower level			x	x	x		
Rowe Score							
preop	5	25	25	5	15	25	16,67
6 weeks	60	60	60	60	60	60	60,00
12 weeks	65	90	90	65	65	90	77,50
6 months	60	100	100	70	90	100	86,67
12 months	90	100	100	90	90	100	95,00
24 months	90	100	100	90	90	100	95,00
36 months	90	100	100	90	90	100	95,00
Re-Dislocations	none	none	none	none	none	none	

Patients were treated with the modLAT, in which the PMFF is used to reconstruct the CAL

*CAL* coracoacromial ligament, *modLAT* modified congruent-arc Latarjet procedure, *PMFF* pectoralis minor fascia flap

### Surgical technique

**Fig. 1:**
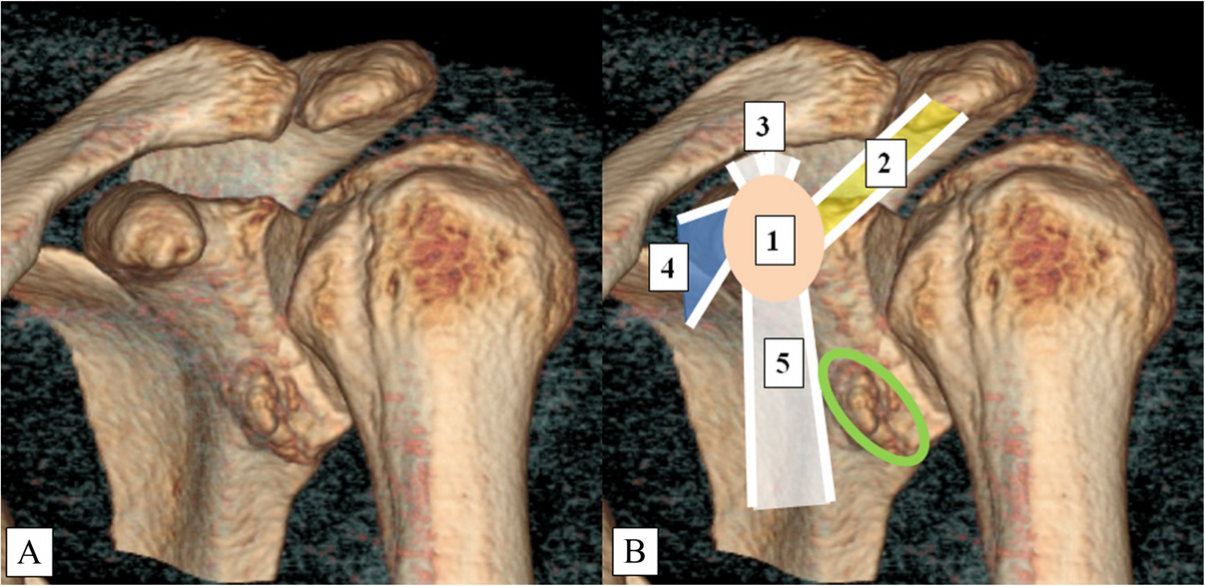
Three-Dimensional Reconstruction of a Computed Tomography Scan of Patient 1 (**a**, **b**). Landmark structures are highlighted (**b**). 1 (beige): CP; 2 (yellow): CAL; 3 (white): CCLs; 4 (blue): PMM; 5 (white): conjoined tendon. The green circle shows the bone defect of the anterior glenoid. CAL, coracoacromial ligament; CCL, coracoclavicular ligament; CP, coracoid process; PMM, pectoralis minor muscle

A detailed preoperative analysis of the functional and patho-anatomical deficits was performed for each patient. In addition to plain radiographs, magnetic resonance imaging (MRI) and computed tomography (CT) with three-dimensional reconstruction was obtained (Fig. 1).

Anteroinferior instability was diagnosed with the appropriate clinical tests in all patients. Patients 1, 4, and 5 showed a bone defect of the anteroinferior glenoid. An example is shown in the CT of patient 1 (Fig. 1). In patient 3, cystic formation around the bone anchors was visible on plain radiographs.

All patients provided written consent to open surgery following a thorough explanation of the surgical technique and the risks and benefits of the procedure as well as of the postoperative rehabilitation protocol.

**Fig. 2:**
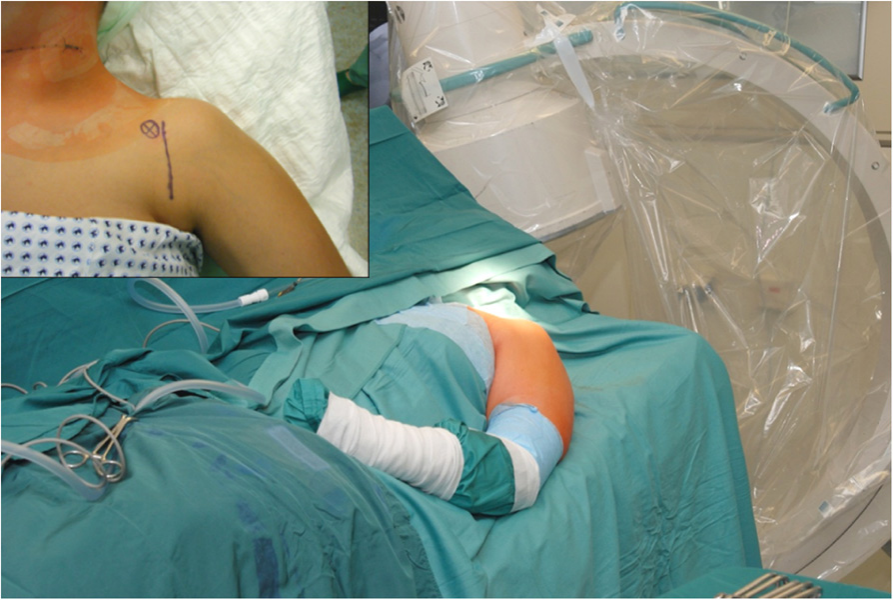
The Semi-Beach-chair Position Used for the Operation. The position must allow true anteroposterior- and axial-view x-rays. The image intensifier is seen in the back. The coracoid process is marked with an X. The skin incision is just lateral to it (insert)

**Fig. 3:**
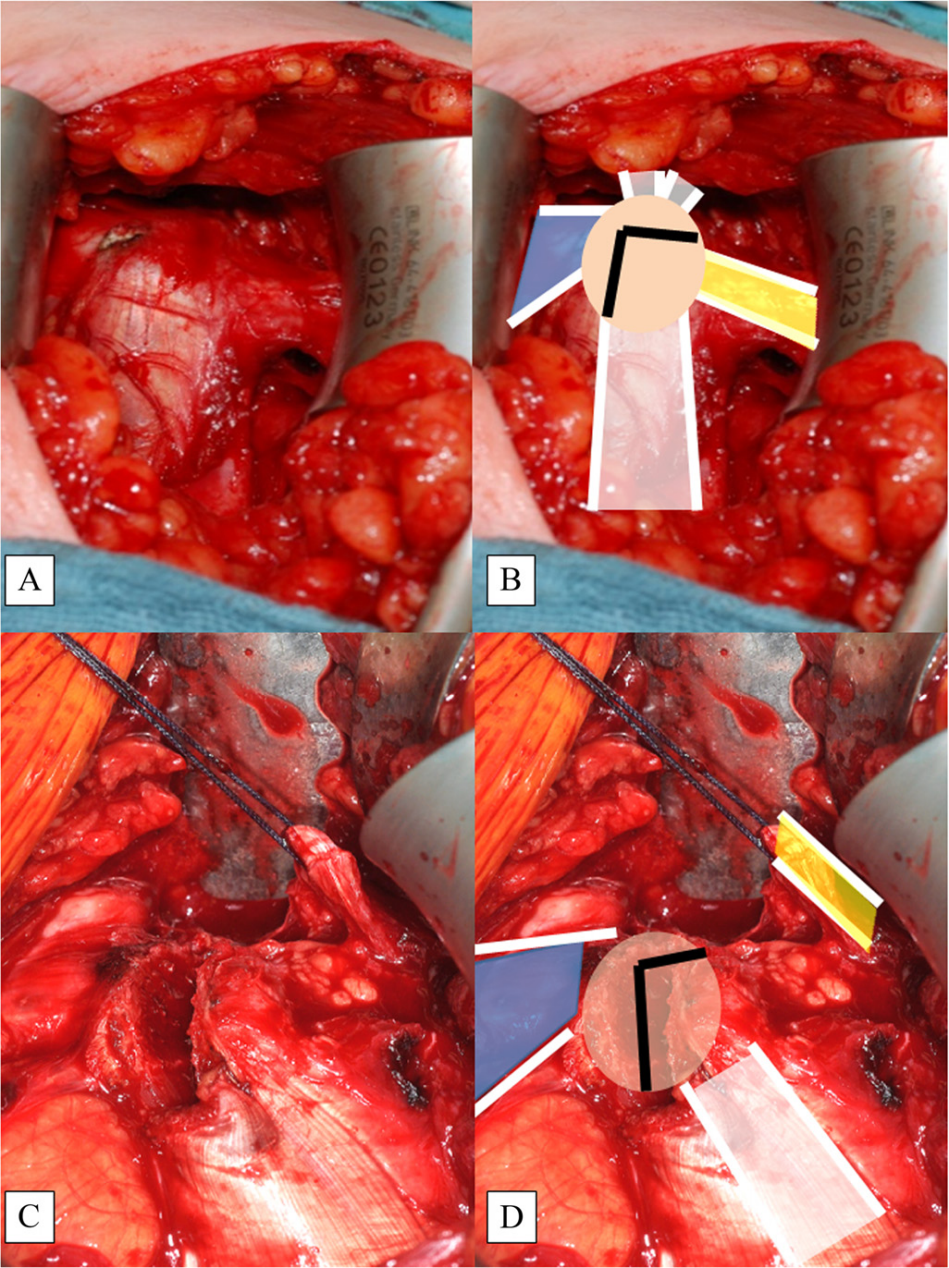
Tip of Coracoid Process (CP, beige) with Attached Structures. Depiction (**a**) and illustration (**b**). The bone cut marked as an inverted L (black lines) (**b**). The L-shaped incision (**c**) and the cut creating the coracoid bone block (**d**) with the conjoined tendon attached (white). Note that the PMM (blue) is attached to the medial one-third of the CP. The CAL (yellow) is secured with a Vicryl™ suture

Surgery was performed under general anesthesia. In addition, a scalene block was administered. The patient was placed in a semi-beach chair position. The landmarks were identified and the skin incision was marked (Fig. 2). The operative technique is as follows:

After skin incision and identification of the deltopectoral interval, the cephalic vein is protected laterally with the self-retractor. The clavipectoral fascia is now visible. The CP is the central landmark, from which the coracoclavicular ligaments (CCLs) and the CAL extend cranially and laterally, respectively. Two important muscular insertions, the pectoralis minor muscle (PMM) and the conjoined tendon, extend medially and caudally, respectively (Fig. 3a, b). In the interval between the PMM and conjoined tendon, the musculocutaneous nerve can be identified and must be protected. Next, an L-shaped incision of the periosteum of the coracoid process is made with the electric knife. This is important for cutting the coracoid bone block. It is necessary to leave about one-third of the CP intact medially, so that the insertion of the PMM is protected (Fig. 3c, d). Before the bone block (lateral two-thirds of the CP) is cut with a small oscillating saw, the periosteal layer must be prepared carefully to enhance the CAL and obtain maximal length. The CAL (with the periosteal insertion) is then secured with a Vicryl™ (Ethicon, Inc., Somerville, NJ, USA) suture (Fig. 3c, d).

**Fig. 4:**
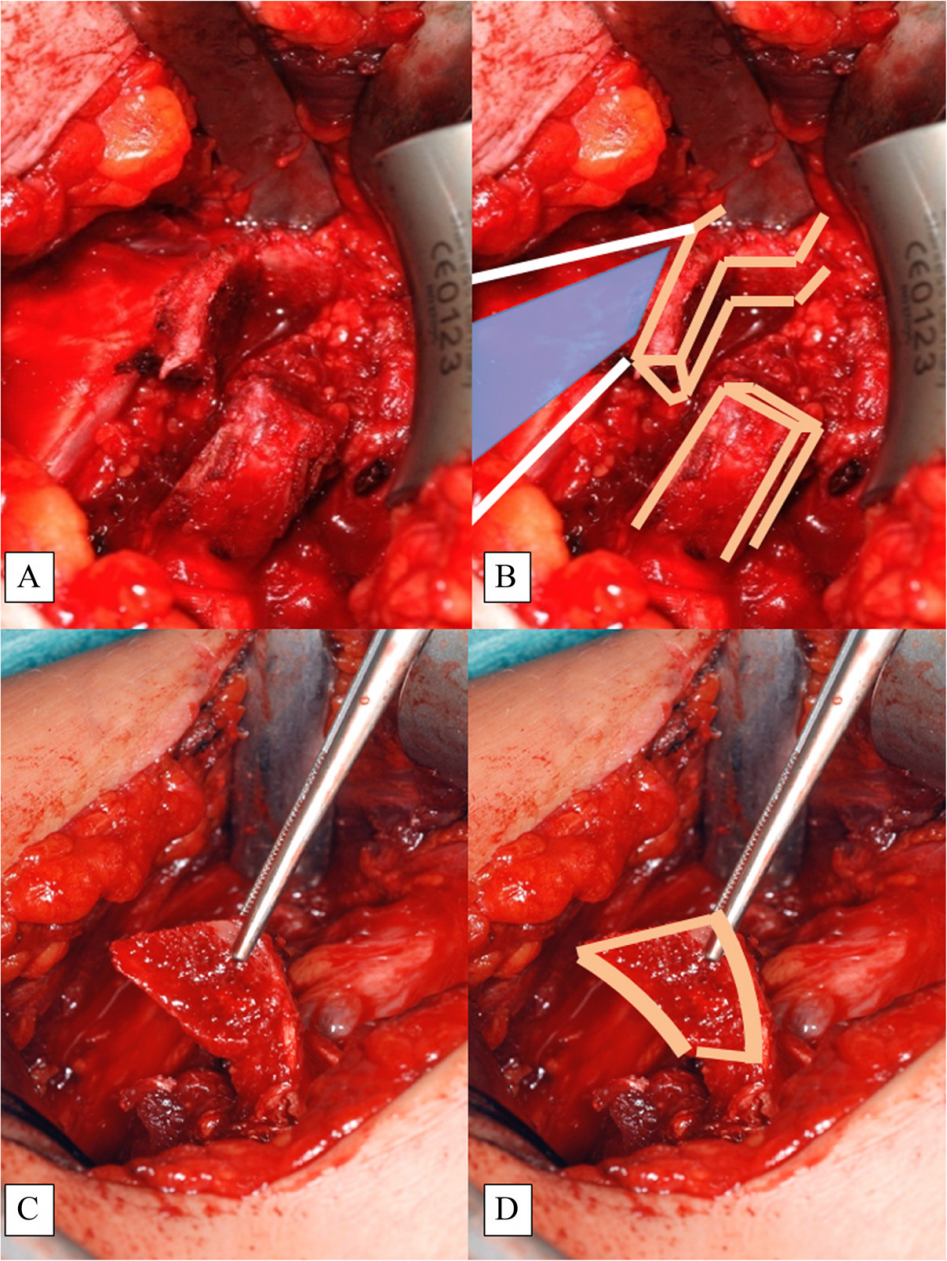
Cutting the Coracoid Bone Block (**a**, **b**) and Identification of the Concave Undersurface (**c**, **d**)

The coracoid bone block is about two-thirds to three-fourths the width of the CP and should be about 2 cm in length (Fig. 4a, b). The undersurface of the block is concave (Fig. 4c, d) and resembles the slightly concave shape of the glenoid. Therefore, the CP is turned 90° around the longitudinal axis so that the undersurface of the CP bone block faces the glenoid surface and the medial cut plane of the CP bone block will be attached to the anterior glenoid vault and fixed with two cannulated screws.

**Fig. 5:**
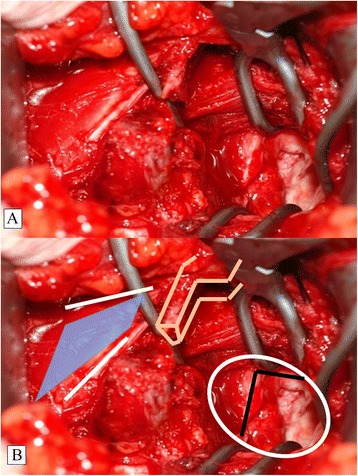
Subscapularis Split at the Level of the Equator of the Glenoid. A K-wire may be used to identify the equator under x-ray control. Special rectangular retractors are required (**a**). The landmarks (PMM and the coracoid stump) and the SSS are shown (**b**). Here, the inverted L-shaped capsular incision is shown in the white oval. PMM, pectoralis minor muscle; SSS, subscapularis split

**Fig. 6:**
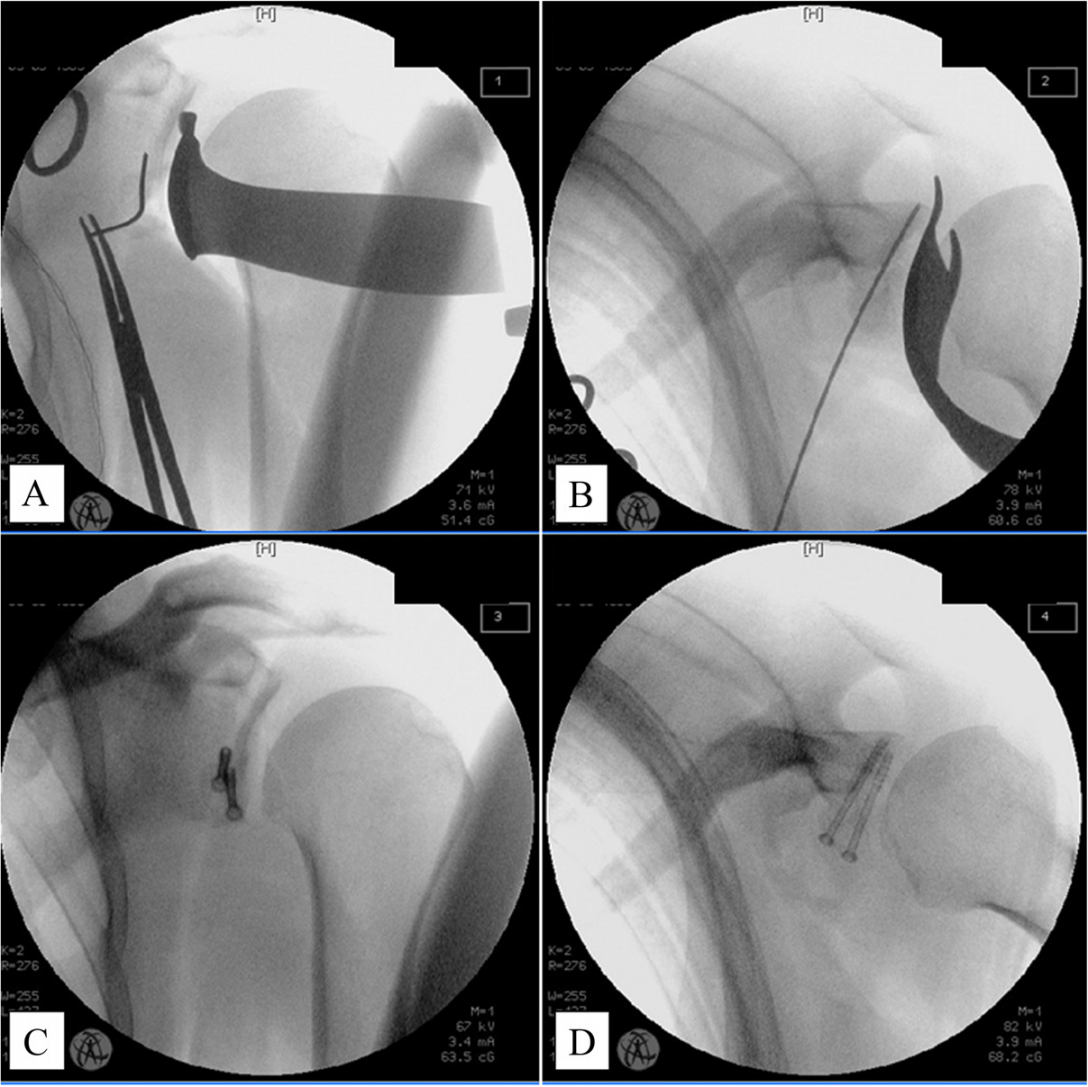
True AP (**a**) and Axial (**b**) Views. Correct screw position and length are indicated by a K-wire. The coracoid bone block is fixed to the glenoid (**c**, **d**). AP, anteroposterior

**Fig. 7:**
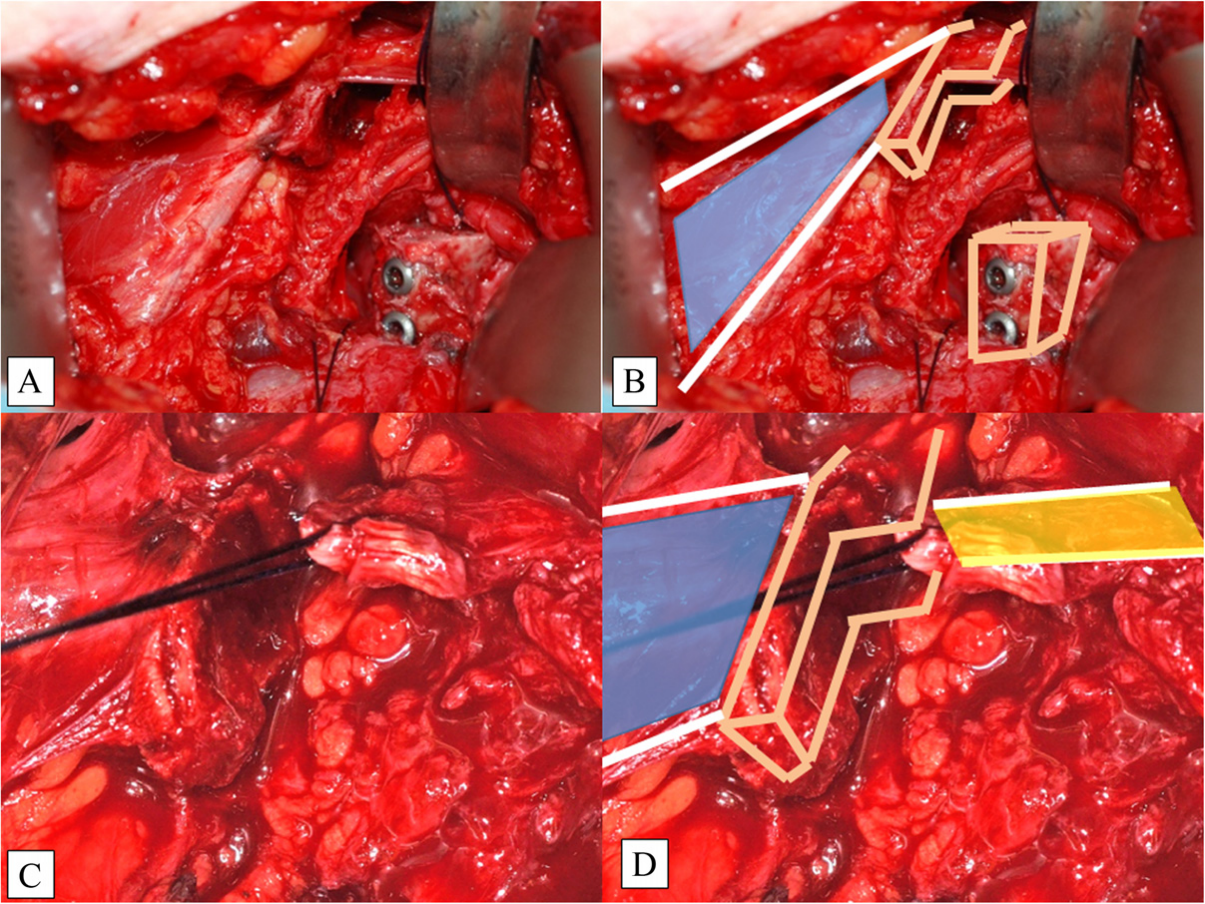
Fixation of the Coracoid Bone Block (**a**, **b**). Note that the PMM (blue illustration) is still attached to the coracoid stump. Closure of the capsule (**c**, **d**). The CAL (yellow illustration) is secured with a strong Vicryl™ suture

In the next step, a horizontal split of the subscapularis muscle is performed longitudinally along the muscle fibers at the level of the equator of the glenoid (Fig. 5). The joint capsule is incised in an inverted L with the long arm parallel to the glenoid rim. The short arm of the L is directed laterally at the level of the equator (Fig. 5b). This is important for capsular plication after the coracoid bone block has been fixed to the glenoid (Figs. 6 and 7). For stable bony fixation, the periosteum at the anterior glenoid vault needs to be removed and the cortical bone slightly abraded; e.g., with a small burr.

It is of utmost importance to get good visualization during the next steps, in which the coracoid bone block is positioned just below the glenoid equator and fixed to the anterior glenoid vault with two cannulated 3.5-mm cancellous screws. This requires an intraoperative x-ray with image intensification. The appropriate planes are shown in Fig. 6.

The coracoid bone block needs to sit flush with, or even better, 1 mm medial to, the anterior glenoid vault (Fig. 7a, b). To increase stability, the posteroinferior capsule is plicated over the coracoid bone block onto the posterosuperior capsule with strong Vicryl™ or PDS™ (Ethicon, Inc.) sutures (Fig. 7c, d).

**Fig. 8:**
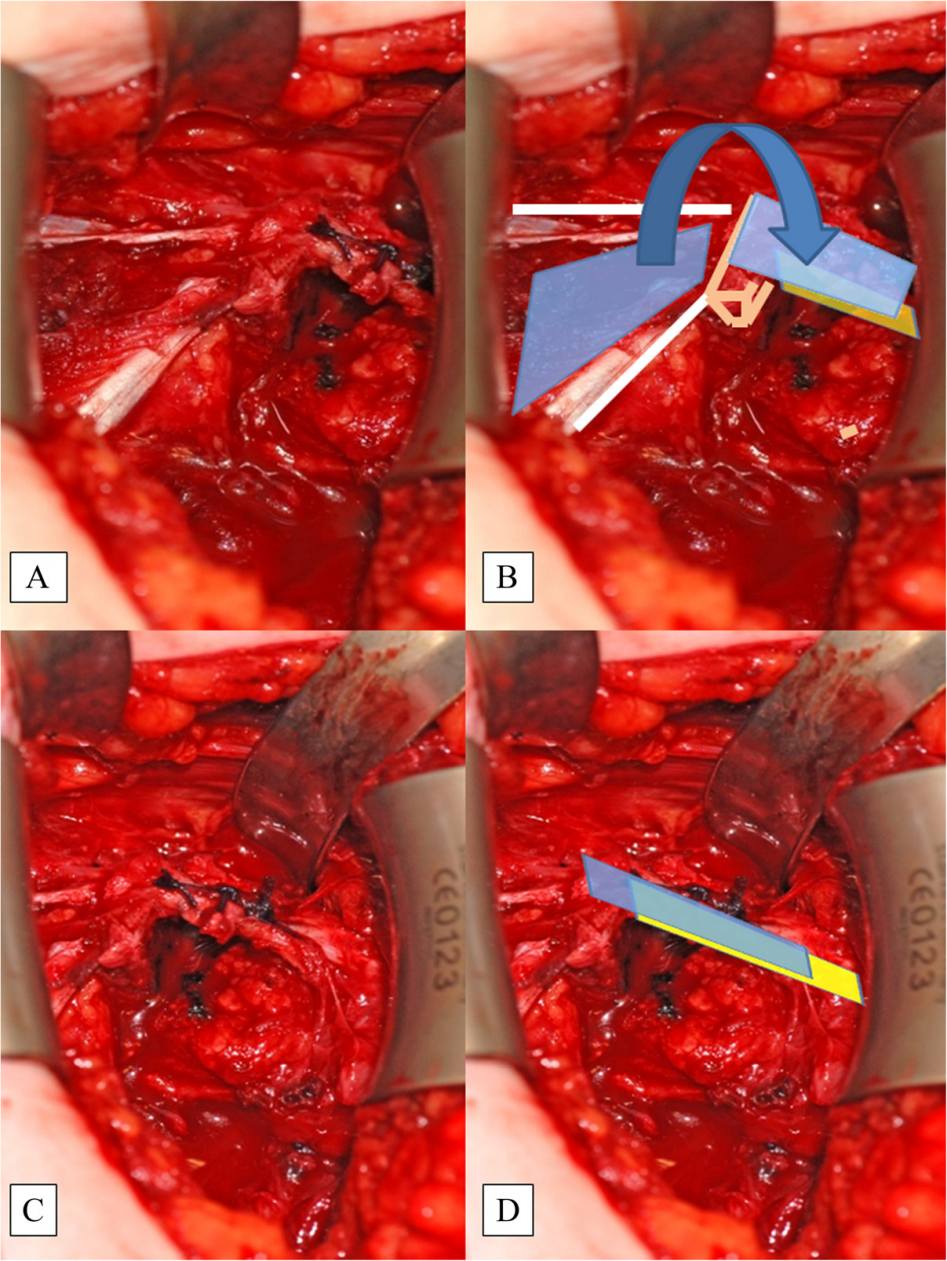
The PMM Fascia (blue illustration) is Incised and Flipped Laterally (**a**, **b**). The CAL (yellow illustration) is stitched to the PMM with strong Vicryl™ sutures (**c**, **d**). This is called the pectoralis minor fascia flap. CAL, coracoacromial ligament; PMFF, pectoralis minor fascia flap; PMM, pectoralis minor muscle

For reconstruction of the CAL, the PMM fascia is incised longitudinally for about 5 cm and a triangular shape created with a medial base of about 2–3 cm. The fascia is carefully detached from the muscle and is left attached distal to the coracoid. This PMFF is flipped over to the lateral side, where it is attached to the stump of the CAL (Fig. 8). The PMFF is usually wide enough to be doubled, strengthening the structure. The PMFF and CAL are stitched together with strong Vicryl™ sutures with maximal overlap and tension.

After removal of all retractors, a drain is placed underneath the pectoralis major muscle. The deltopectoral interval is attached and the cephalic vein is checked. The skin is closed and the wound dressed in the usual fashion.

### Postoperative care and follow-up examination

In our six patients, the arm was immobilized in adduction and internal rotation in a Gilchrist shoulder brace for 10 days, followed by a more comfortable shoulder sling. Physiotherapy started the day after surgery with pendulum exercises, followed by a 6-week rehabilitation program with 2–3 sessions per week in an outpatient setting. Postoperatively, the range of motion of the glenohumeral joint was measured in all planes. At 3, 6, 12, 24, and 36 months, the stability of the shoulder was tested using the apprehension test and the sulcus sign. Three parameters—shoulder stability, motion, and function—were evaluated using the 1978 version of the Rowe score [16]. Surgical outcome was classified as poor (final score ≤ 50 points), fair (51–74 points), good (75–89 points), or excellent (90–100 points).

Data were collected using an Excel spreadsheet. Because of the small number of patients in this case series, statistical analysis was not performed.

## Results

All six patients underwent modLAT with CAL reconstruction using a PMFF. Operative duration ranged from 78 to 112 min (mean 94.83 min). None of the patients had intra- or postoperative complications related to the surgical procedure. Most importantly, no patient had re-dislocation of the surgically treated shoulder during follow-up. All patients returned to their preoperative sports activity. Of the six patients, the three who had performed shoulder-intensive sports (canoeing, handball, and swimming) competitively preoperatively chose to perform their activity at a non-competitive level.

Functional outcome as measured by the Rowe score [16] increased from 16.67 points preoperatively to 77.5 points 12 weeks after surgery. At 12 months, Rowe score plateaued at 95 points, remaining at this level until the final follow-up at 36 months (Table 1). Interestingly, all three patients who reached 100 points postoperatively had no bony defect of the anterior glenoid and had started with an above-average preoperative Rowe score of 25 points.

## Discussion

The effect of the CAL on superior shoulder translation has been demonstrated in several biomechanical studies; however, these have focused largely on the effect in rotator cuff-deficient shoulders or those with symptoms of impingement [2–5, 7].

A recent study indicates that performing a Latarjet procedure can lead to an increase in superior shoulder translation in most joint configurations and loading conditions [8]. This highlights the importance of the CAL as a restraint to superior humeral head translation, even in patients with an intact rotator cuff.

There are some reports about regeneration of the CAL [17, 18]. However, these studies were performed in cases of acromioplasty and arthroscopic subacromial decompression. Therefore, it seems important to maintain the integrity of the CAL whenever possible.

The classic Latarjet procedure of coracoid transfer was later modified by Helfet [19], who named it for his mentor, Rowley Bristow. The aim of these procedures is to stabilize the shoulder with the static action of the transferred bone block and the attached conjoined tendon. In the classic Latarjet procedure, the CP is osteotomized posteriorly at the junction of its horizontal and vertical parts and then transferred. Only the tip of the CP is transferred in the Bristow procedure, whereas in the classic Latarjet procedure, the transfer includes a portion of the CAL, which is sutured to the capsular tissue through a short horizontal incision made in the subscapularis. The Latarjet procedure reconstructs the glenoid depth and width with the bone block and creates dynamic reinforcement of the inferior part of the capsule through the conjoined tendon, particularly when the arm is abducted and externally rotated. Transferring the CP does provide reliable and durable stabilization of the shoulder [20–24].

De Beer developed the congruent-arc modification of the classic Latarjet, which rotates the coracoid graft 90° to its position in the classic Latarjet procedure [13]. This has several advantages, including a radius of curvature matched to that of the glenoid and the ability to reconstitute greater glenoid bone loss. Because of the matching radius of curvature and the potential for greater bony conformity and constraint, it is conceivable that the CP oriented in a congruent manner could decrease superior humeral head translation. The study by Degen et al. [8], however, indicates that the congruent-arc modification and the classic Latarjet did not differ significantly (*P* > 0.05) with regard to superior translation.

The new technique presented here aims to reconstruct the CAL as an important structure of the coracoacromial arc and thereby prevent superior humeral head translation. Based on the congruent-arc Latarjet, some additional modifications were developed, especially with regard to the preparation of the CAL.

The CP is cut in an L-shaped fashion so that the PMM stays attached medially to the stump of the bone. Because of this, the bone block is about 2 cm long, and the concave undersurface enhances the glenoid, while the cut side with the exposed cancellous bone is fixed to the anterior glenoid vault. Before cutting the bone block, the CAL is detached from the CP medially by careful subperiosteal preparation of the lateral two-thirds of the CP. This is important to derive maximal length of the CAL for later reconstruction with the PMFF. For this, a 3 × 5-cm portion of the PMM fasciae is prepared from the muscle but left attached medially at the coracoid stump. The fascia is flipped over laterally and carefully stitched onto the CAL.

A limitation of this study is its relatively small sample size, which is consistent with other small case series. Further studies are needed to determine if this method is sufficient to prevent superior shoulder translation following Latarjet coracoid transfer in a clinical experimental clinical setting.

One potential complication is fracture of the CP during and after surgery, especially in the case of a small CP, such as in female and younger patients. The size of the CP varies among patients according to physical activity and body size. To avoid fracture of the CP, no more than three-fourths of its width should be cut. However, because the PMFF is flipped and stitched to the stump of the CAL, the PMFF acts as a dynamic restraint and stabilizes the CP.

## Conclusion

The classic and congruent-arc Latarjet procedures, which disrupt the CAL, increase superior humeral head translation and possibly anterior glenohumeral instability. Reconstruction of the CAL using the PMFF is a safe method that restores the coracoacromial arc and may improve shoulder function in patients undergoing a Latarjet procedure.
